# A NMR-Based Metabolomic Approach to Investigate the Antitumor Effects of the Novel [Pt(*η*^1^-C_2_H_4_OMe)(DMSO)(phen)]^+^ (phen = 1,10-Phenanthroline) Compound on Neuroblastoma Cancer Cells

**DOI:** 10.1155/2022/8932137

**Published:** 2022-06-10

**Authors:** Federica De Castro, Erika Stefàno, Erik De Luca, Antonella Muscella, Santo Marsigliante, Michele Benedetti, Francesco Paolo Fanizzi

**Affiliations:** Department of Biological and Environmental Sciences and Technologies (DiSTeBA), University of Salento, Via Monteroni, I-73100 Lecce, Italy

## Abstract

NMR-based metabolomics is a very effective tool to assess the tumor response to drugs by providing insights for their mode of action. Recently, a novel Pt(II) complex, [Pt(ƞ^1^-C_2_H_4_OMe)(DMSO)(phen)]^+^ (phen =  1,10-phenanthroline), Pt-EtOMeSOphen, was synthesized and studied for its antitumor activity against eight human cancer cell lines. Pt-EtOMeSOphen showed higher cytotoxic effects than cisplatin in most of the cancer cell lines and in particular against the neuroblastoma cell line (SH-SY5Y). In this study, the mechanism of action of Pt-EtOMeSOphen on SH-SY5Y cells was investigated using ^1^H NMR-based metabolomics and compared with cisplatin. The observed time response of SH-SY5Y cells under treatment revealed a faster action of Pt-EtOMeSOphen compared with cisplatin, with a response already observed after six hours of exposure, suggesting a cytosolic target. NMR-based metabolomics demonstrated a peculiar alteration of the glutathione metabolism pathway and the diacylglycerol expression.

## 1. Introduction

Cancer still remains one of the major causes of death worldwide with chemotherapy, radiation, and surgery representing the common therapeutic treatment of oncological patients [1]. Among these, chemotherapy constitutes the most significant treatment and cisplatin is one of the most efficient and globally used antitumor drugs [2, 3]. However, the side effects and resistance phenomena related to the use of cisplatin triggered many researchers toward the development of new platinum-based antitumor drugs [4–11], even finding other interesting properties and possible therapeutic applications [12]. Among the investigated new platinum species, organometallic compounds (OCs) have been recently considered as promising anticancer drugs. These metal complexes are characterized by the presence of at least one direct covalent metal-carbon bond in their structure. The presence of a coordinating central metal ion allows greater structural diversity (from linear to octahedral), thus a more flexible stereochemistry compared with organic compounds. Interestingly, this specific feature can also provide a better control of kinetic properties, such as the hydrolysis rate of ligands [13]. The marked differences of OCs from the “classical” coordination metal complexes together with their specific pharmacological potential render this type of complex very attractive for new drug design. On the other hand, only a limited number of studies on platinum OCs tested as antitumor drugs were performed [13], suggesting further commitment in the field.

Many research projects have demonstrated cancer cells have a reprogrammed and specific metabolism that is very different from healthy cells [14–22]. For this reason, the study of tumors at the molecular level using a metabolomic approach is a useful tool for drug development. NMR spectroscopy has emerged as a critical tool for metabolomics in drug discovery and is defined a “gold standard method” for medical and pharmacological studies [23]. In the last few years, NMR-based metabolomics has been extended to the study of metal-based antitumor drugs for assessing mode of action and specific targets in cancer cells [24, 25]. The application of NMR spectroscopy in this research field opened new perspectives for the study of new potential drugs by providing a metabolic fingerprint of biological systems and simplifying preclinical trials [24–28].

**Figure 1:**
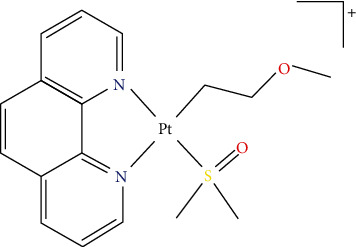
Chemical structure of the novel [Pt(ƞ^1^-C_2_H_4_OMe)(DMSO)(phen)]^+^ organometallic Pt(II) complex, Pt-EtOMeSOphen.

In these contexts, we have recently reported the synthesis and the cytotoxic activity of a novel Pt(II) organometallic complex of the type [Pt(ƞ^1^-C_2_H_4_OMe)(DMSO)(phen)]^+^ (Pt-EtOMeSOphen) (Figure 1) [29].

The cytotoxic activity of Pt-EtOMeSOphen has been evaluated on different cancer cells lines (SH-SY5Y, SK-OV-3, Hep-G2, Caco-2, HeLa, MCF-7, MG-63, ZL-55), showing a higher cytotoxicity compared with cisplatin in many of the tested cell lines and in particular against the neuroblastoma cell line SH-SY5Y. Moreover, we have recently demonstrated that Pt-EtOMeSOphen is able to inhibit SH-SY5Y cancer cells' survival, motility, and invasion [29].

In this work, a ^1^H NMR metabolomic approach was used to explore, in comparison with cisplatin, the possible mechanisms of action and to define the relevant targets of Pt-EtOMeSOphen on neuroblastoma model cancer cells (SH-SY5Y).

## 2. Materials and Methods

### 2.1. Synthesis of Complexes

All solvents and reagents were purchased from Aldrich Chemical Company and used as received, except otherwise stated. Cisplatin, *cis*-[PtCl_2_(NH_3_)_2_], and Pt-EtOMeSOphen were synthesized according to previously reported procedures and gave satisfactory analytical data [29, 30].

### 2.2. Cell Cultures and Treatments

SH-SY5Y cells were cultured in 1 : 1 mixture of DMEM (high glucose) and Ham's F-12 Nutrient Mixture (Sigma, St. Louis, MO, USA) supplemented with 10% heat-inactivated fetal bovine serum (FBS), glutamine 2 mM, penicillin (100 U/mL), and streptomycin (100 mg/mL) in a humidified incubator containing 5% CO_2_ in air at 37°C. Cells were grown to 70–80% confluence and then treated with the half maximal inhibitor concentration (IC_50_) at 24 h of cisplatin and Pt-EtOMeSOphen (50 *μ*Μ) for different incubation periods (6, 12, and 24 h). For control cells, fresh medium without drug was added. Then, cells were harvested by trypsinization, washed with PBS, and pelleted by centrifugation (1000 rpm × 10 min). The same procedure was rigorously used for all samples to minimize experimental variability. For each condition, three independent assays were performed. A total of 36 samples were analyzed.

### 2.3. Cell Sampling for NMR Analysis

NMR samples were prepared from pelleted SH-SY5Y cells and from each respective recovered culture medium. A combined extraction of polar and lipophilic metabolites from cell pellets, using a methanol/chloroform/water mixture, was carried out, according to reported literature [31]. Each sample was stored at −80°C until analysis.

The preparation of samples was carefully carried out following a specific protocol in order to minimize the possible NMR chemical shift variations in the subsequent analysis, according to reported literature [26–28, 31].

In detail, each polar cell extract was resuspended in 580 *µ*L NMR buffer (0.1 M K_2_HPO_4_, 0.2 mM TSP, 2 mM NaN_3_), pH 7.4, in D_2_O. Samples were vortexed and centrifuged at 12,000 *g* for 5 min at 4°C to remove any solid debris, and 550 *μ*L of the supernatant was transferred into 5 mm NMR tubes. The lipid cell extracts were resuspended in 580 *μ*L of deuterated solvent (CDCl_3_) containing 0.03 vol/vol TMS, then vortexed, and centrifuged (1000*g* for 5 min), and 550 *μ*L of the supernatant was transferred into 5 mm NMR tubes. For culture media, 900 *μ*L of each sample was added with 100 *μ*L of NMR buffer (1.5 M K_2_HPO_4_, 2 mM TSP, 2 mM NaN_3_), pH 7.4, in D_2_O. Samples were vortexed, and 600 *μ*L of the supernatant was placed in 5 mm outer diameter NMR tubes, as reported [26, 27, 31].

### 2.4. NMR Measurements

All measurements were performed on a Bruker Avance III 600 Ascend NMR spectrometer (Bruker, Ettlingen, Germany), operating at 600.13 MHz for ^1^H observation, equipped with a TCI cryoprobe (triple resonance inverse cryoprobe) incorporating a *z*-axis gradient coil and automatic tuning matching (ATM). Experiments were acquired at 300 K in automation mode after loading each sample on a Bruker automatic sample changer, interfaced with the software IconNMR (Bruker).

For each aqueous and culture media sample, a standard 1D ^1^H spectrum with presaturation and composite pulse for selection (ZGCPPR Bruker standard pulse sequence) and Carr–Purcell–Meiboom–Gill (CMPG) spin-echo sequence was acquired with 64 transients, 16 dummy scans, 5-s relaxation delay, size of FID (free induction decay) of 32 K data points and zero-filling by a factor of 2 to give 64 K frequency domain data points, P1 11.38 *μ*s, a spectral width of 12,019.230 Hz (20.0276 ppm), an acquisition time of 1.36 s, a total spin-spin relaxation delay of 1.2 ms, and solvent signal saturation during the relaxation delay. The resulting FIDs were multiplied by an exponential weighting function corresponding to a line broadening of 0.3 Hz before Fourier transformation, automated phasing, and baseline correction. All spectra were referenced to the trimethylsilyl propionate (TSP) signal (*δ* = 0.00 ppm).

For lipid extracts, a one-dimensional experiment (ZG experiment) was run with 64 scans, 16 dummy scans, 5-s relaxation delay, 64 K time domain, spectral width 20.0276 ppm (12019.230 Hz), and P1 8 *μ*s. All spectra were referenced to the tetramethylsilane (TMS) signal (*δ* = 0.00 ppm).

Cell extracts are multicomponent systems; for this reason, for all samples, the NMR characterization of the extracts was performed by using 1D (^1^H) and 2D (^1^H-^1^H J-resolved, ^1^H-^1^H COSY correlation spectroscopy) NMR spectra. Moreover, ^1^H-^13^C HSQC (heteronuclear single quantum correlation) and ^1^H-^13^C HMBC (heteronuclear multiple bond correlation) were randomly acquired for metabolite assignment purposes. Metabolite identification was carried out from 1D and 2D NMR profiles using associated databases of pure compounds such as Human Metabolome Database (HMDB) and ChenomX NMR Suite 8 (ChenomX Inc., Edmonton, Canada) software and compared with other published data [32]. To evaluate the presence of variations in metabolites' chemical shifts (of aqueous and lipid extracts and of culture media), in the prebucketing processing, the alignment of the acquired spectra was performed. No variations were observed confirming the reliability of the NMR analysis for the subsequent buckets processing and multivariate data analysis. The NMR spectra were processed for the visual inspection and the bucketing process using Topspin 3.6.1 and Amix 3.9.13 (Bruker, Biospin, Italy).

### 2.5. Data Processing and Multivariate Data Analysis

The ^1^H NMR chemical shifts for detected metabolites are reported in Tables S1-S2. The bucketing preprocessing procedure was applied on the CMPG spectra, for both aqueous extracts and culture media, and on the ZG spectra, for lipid extracts, covering the range 10.0–0.5 ppm. Each NMR spectrum was automatically divided in rectangular buckets of fixed 0.01 ppm width and integrated using Bruker Amix 3.9.13 software (Bruker, Biospin) software. The spectral regions between 5.10 and 4.7 ppm (containing the residual peak from the suppressed water resonance) for aqueous extracts and culture media and between 7.60 and 7.00 and 3.60 and 3.00 ppm (containing signals of chloroform and its carbon satellites and the residual methanol, respectively) for lipid extracts were excluded. The remaining buckets were normalized to total area to minimize small differences and subsequently mean-centered. Multivariate statistical analyses (unsupervised principal component analysis (PCA) and the supervised orthogonal partial least squares discriminant analyses (OPLS-DA)) were performed to examine the intrinsic variation in the data using SIMCA 14 software (Sartorius Stedim Biotech, Umeå, Sweden) [33–35]. The Pareto scaling procedure was performed by dividing the mean-centered data by the square root of the standard deviation [14, 15]. The robustness of the statistical models was tested by the cross-validation default method (7-fold) and further evaluated with a permutation test (100 permutations) [35]. The total variations in the data and the internal cross-validation, thus the quality of the statistical models, were described by *R*^2^ (cum), *Q*^2^ (cum) parameters, and *p* values (p [CV-ANOVA], a *p*-value <0.05, confidence level of 95%, was considered statistically significant) obtained from the analysis of variance testing of cross-validated predictive residuals (CV-ANOVA) [15, 16, 31, 33].

The variation in the metabolites content between the two different conditions of treatment (Pt-EtOMeSOphen, cisplatin) were calculated as the −log_2_ fold change (FC) ratio of the normalized median intensity for the distinctive metabolites in the spectra, identified by OPLS-DA analysis, with respect to controls. The Box-and-Whisker plots illustrating the trend of significant metabolites for Pt-EtOMeSOphen at six hours of treatment were obtained by using the biomarker analysis on MetaboAnalyst software. The statistical significance was evaluated using a two-sample *t*-test considering *p*-values <0.05 as statistically significant.

### 2.6. Metabolic Pathway Analysis

The most relevant metabolic pathways potentially involved in the metabolomic study were identified using MetaboAnalyst software [17, 18]. The aim was to investigate if certain metabolic pathways are significantly different in the treated SH-SY5Y aqueous extracts (all condition of treatment) when compared with control samples. Metabolites of interest previously quantified by selected distinctive unbiased NMR signals were used as the input matrix for the metabolic pathway analysis. The pathway impact was calculated as the sum of the importance measures of the matched metabolites normalized by the sum of the importance measures of all metabolites in each pathway.

## 3. Results

### 3.1. Endo- and Exo-Metabolome ^1^H NMR Profiling of SH-SY5Y Cells and Unsupervised Principal Components Analysis (PCA)

In the present study, a ^1^H NMR metabolomic analysis of cell lysates (aqueous and lipid extracts) and their growth media was performed to study the metabolic effects of Pt-EtOMeSOphen treatment on SH-SY5Y cells. The cells were treated (for 6, 12, and 24 h) with Pt-EtOMeSOphen 50 *μ*M, concentration corresponding to the IC_50_ value for SH-SY5Y cells at 24 h [29]. Moreover, a further parallel assay with cisplatin using the same experimental setup and design (the cisplatin IC_50_ value and same times of cells treatment) was performed. Both treatments (Pt-EtOMeSOphen and cisplatin) were also compared with untreated control cells.

**Figure 2:**
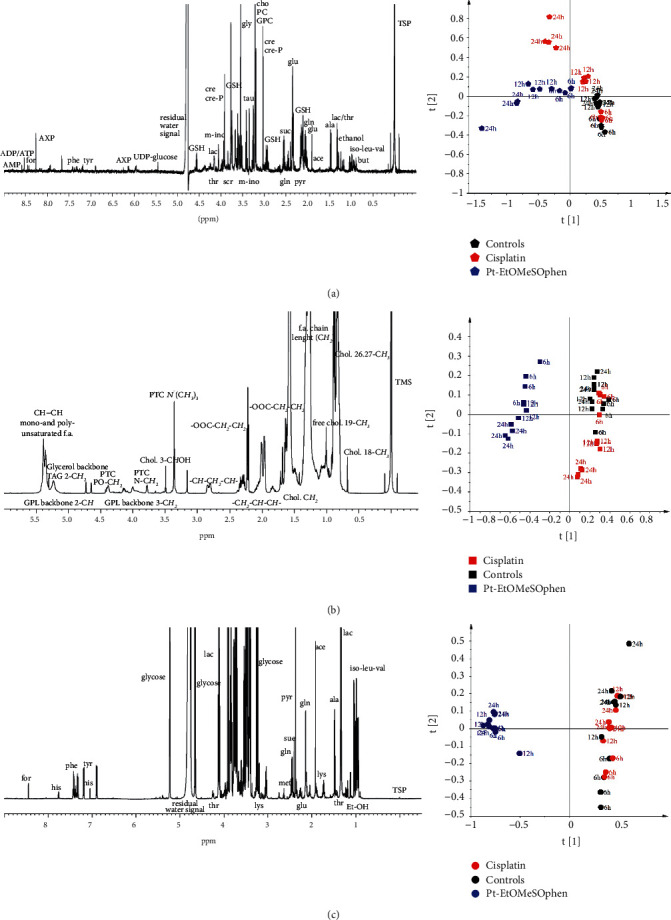
Representatives 600 MHz ^1^H NMR spectra (left panels) and corresponding PCA score plot models (right panels) of SH-SY5Y cells: (a) aqueous extracts (*R*^2^*X* = 0.566; *Q*^2^ = 0.297); (b) lipid extracts (*R*^2^*X* = 0.946; *Q*^2^ = 0.913); and (c) growth media (*R*^2^*X* = 0.879; *Q*^2^ = 0.851). Iso: isoleucine, leu: leucine, val: valine, but: butyrate, lac: lactate, thr: threonine, ala: alanine, ace: acetate, glu: glutamate, gln: glutamine, GSH: glutathione, lys: lysine, for: formate, met: methionine, pyr: pyruvate, suc: succinate, cre: creatine, cre-P: creatine phosphate, cho: choline, PC: phosphocholine, GPC: glycerophosphocholine, m-ino: myo-inositol, phe: phenylalanine, tyr: tyrosine, his: histidine, tau: taurine, gly: glycine, ser: serine, UDP-glucose: uridine diphosphate glucose, AXP: adenosine mono- (M), di- (D), triphosphate (T), PTC: phosphatidylcholine, GPL: glycerophospholipids.

Figure 2 (left panels) shows the 600 MHz ^1^H NMR spectra obtained from the aqueous extract (A), lipid extract (B), and growth media (C) of SH-SY5Y cells. The expansion of the NMR spectra is available in Figures S1-S3. The ^1^H NMR resonances assignment is listed in Tables S1 and S2.

The aqueous extract (Figure 2(a)) was constituted by amino acids (isoleucine, valine, leucine, alanine, glutamine, glutamate, tyrosine, phenylalanine, glycine, serine, threonine, creatine, and creatine phosphate), osmolytes (free choline, phosphocholine, glycerophosphocholine, and taurine), organic acids (acetate, pyruvate, succinate, formate), inositol (myo-inositol), and nucleoside derivatives (AMP, ADP, ATP, UDP-glucose) (Table S1).

The lipid fraction (Figure 2(b)) was characterized by the resonances from protons of fatty acids, phosphatidylcholine, mono- and polyunsaturated lipids, and mono- and triglycerides (Table S2). Interestingly, only in the lipid fraction of the Pt-EtOMeSOphen-treated cells was found also the resonances ascribable to diglycerides.

Nutrient substrates essential for cell growth such as amino acids (isoleucine, valine, leucine, tyrosine, histidine, lysine, glutamine, phenylalanine, alanine, glycine, and glutamate) and sugars (glucose) characterize the culture medium NMR spectra (Figure 2(c)). Moreover, other specific metabolic intermediates released in culture media such as glycolysis and TCA cycle compounds (pyruvate, succinate, acetate, lactate) and waste metabolites (formate) were found (Table S1).

In order to investigate the differences between treated cells (Pt-EtOMeSOphen and cisplatin) and controls, at increasing incubation times (6, 12, and 24 h), a preliminary multivariate data analysis was performed. Bucket tables obtained using the cell growth media and lysate aqueous extracts (^1^H CPMG NMR spectra) and cell lipid extracts (^1^H ZG NMR spectra) were used as input variables.

The PCA showed for both endo- (intracellular) and exo- (culture medium) metabolome (Figure 2(a)–2(c) right panels) marked changes in the metabolic profile for Pt-EtOMeSOphen treated with respect to untreated and cisplatin-treated SH-SY5Y cells. Moreover, the PCA score plot suggested a drug incubation time dependence of the endo-metabolome profile for both treatments not observed for the controls (dispersion along the PC1 and PC2 for Pt-EtOMeSOphen and cisplatin, respectively). On the other hand, the PCA for the exo-metabolome profiles showed a clear separation along PC1 (but minimal dispersion along both PC1 and PC2 components) for Pt-EtOMeSOphen treated compared with untreated and cisplatin-treated samples (both scattered along the PC2 component) (Figure 2(c) right panel).

In detail in all obtained PCA models (Figure 2(a)–2(c)), only the samples treated with Pt-EtOMeSOphen separated along the PC1 component, with respect to the controls (with a clear time dependence for the endo-metabolome profile). On the contrary, cisplatin-treated samples nearly overlapped with controls at short drug incubation time (6 h) and a clear separation from these latter was only observed later (after 12–24 h) and essentially along the PC2 component. Coherently, the decrease in cell viability of SH-SY5Y cells was already observed after six hours of exposure for Pt-EtOMeSOphen, while twelve hours were required for cisplatin, as reported in our previous studies [19].

### 3.2. Supervised Analysis (OPLS-DA) and Metabolomic Changes

**Figure 3:**
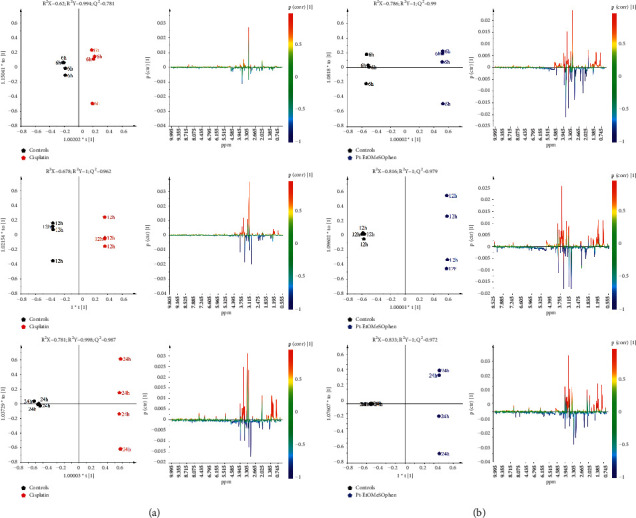
Time response pairwise comparison OPLS-DA score plot models (1 + 2 + 0 components) and corresponding color-coded correlation coefficient S-line plots displaying the related predictive discriminant loadings variables derived from ^1^H CPMG NMR spectra of SH-SY5Y cell aqueous extracts obtained from different pairwise groups. (a) Controls vs. cisplatin. (b) Controls vs. Pt-EtOMeSOphen.

**Figure 4:**
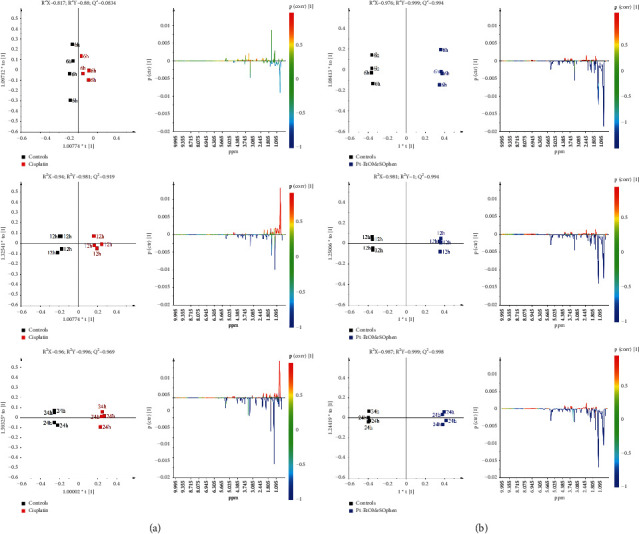
Time response pairwise comparison OPLS-DA score plot models (1 + 2 + 0 components) and corresponding color-coded correlation coefficient S-line plots displaying the related predictive discriminant loadings variables derived from ^1^H NMR spectra of SH-SY5Y cells lipid extracts obtained from different pairwise groups. (a) Controls vs. cisplatin. (b) Controls vs. Pt-EtOMeSOphen.

The supervised orthogonal partial least squares discriminant analysis (OPLS-DA) was used to further investigate the treatment-related (Pt-EtOMeSOphen, cisplatin) metabolic differences with respect to the controls. According to the time dependence suggested by the PCA of the endo-metabolome, the administration times (6, 12, and 24 h) were also considered in the OPLS-DA pairwise comparisons. The time-dependence analysis for the metabolic cellular response to treatments is of crucial importance for understanding the drug action mechanism. Useful information can be gained about the drug-induced early or late cellular pathways alterations. Twelve OPLS-DA models (six for the aqueous and six for the lipid extracts) were obtained for the endo-metabolome analysis of SH-SY5Y cells, using one predictive and two orthogonal components (Figures 3 and 4). In all the cases, the models were characterized by good-quality parameters (*R*^2^ and *Q*^2^), describing the total variations in the data and the predictive capability.

**Figure 5:**
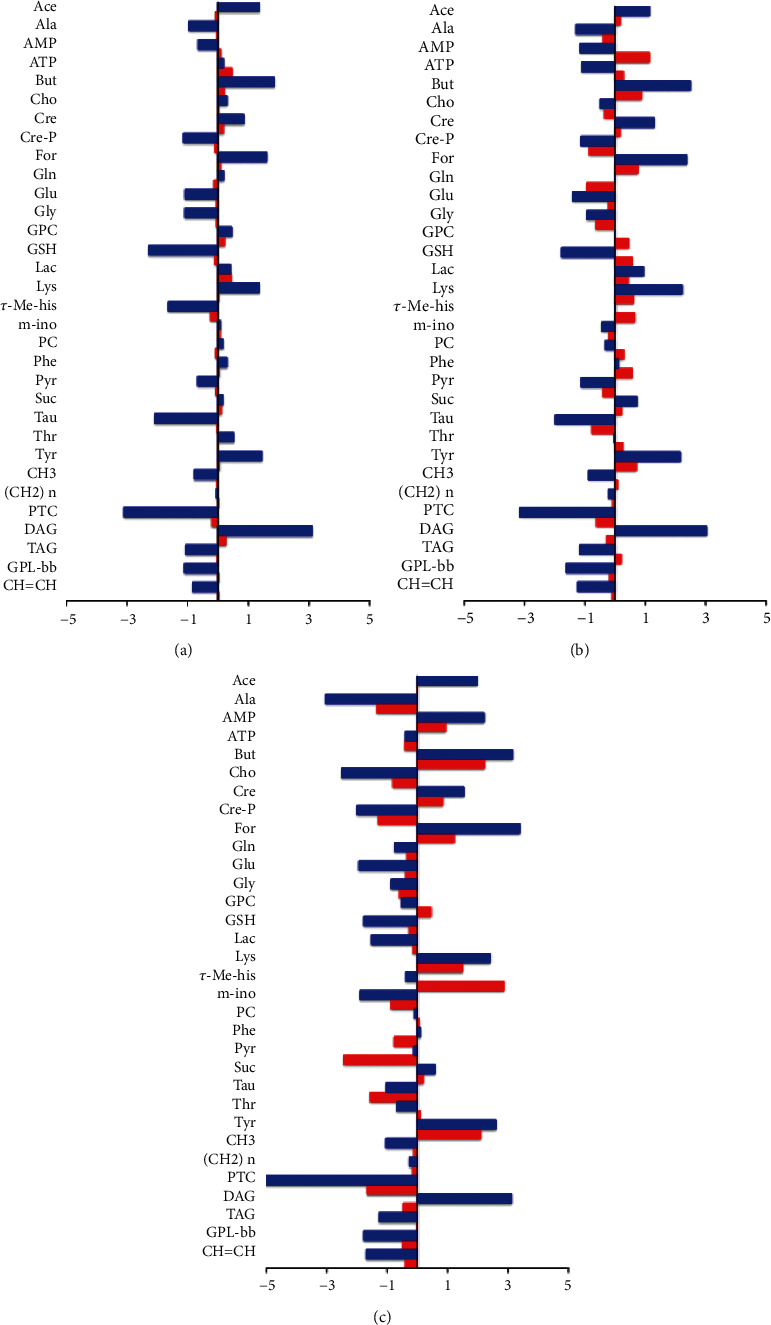
Relevant discriminant metabolites comparison of SH-SY5Y endo-metabolome obtained from different pairwise groups at the different times of treatments: (a) six hours, (b) twelve hours, and (c) twenty-four hours. Blue: Pt-EtOMeSOphen vs. controls; red: cisplatin vs. controls. Metabolites with –Log_2_ (FC) negative values have lower concentration compared with controls. Metabolites with –Log_2_ (FC) positive values have higher concentration compared with controls. ^∗^All the considered FC values have *p* value < 0.05 except for the cisplatin in panel A in which the FCs have *p* value > 0.05.

For all the considered incubation times, the OPLS-DA score plots (Figures 3 and 4) showed a clear separation between treated (Pt-EtOMeSOphen, cisplatin) groups and the control group on the model predictive component. The metabolites responsible for the observed classes discrimination were easily found from the S-line plot for each model. The observed fold change (FC) ratios, for the identified metabolites (Table S1-S2), obtained by comparison of specific representative signal containing buckets, are reported in Figure 5.

Interestingly, faster and greater cellular alterations were induced by Pt-EtOMeSOphen compared with cisplatin. After six hours, SH-SY5Y cells react at Pt-EtOMeSOphen exposure with significant changes in their endo-metabolome expression. In detail, the decrease of the GSH, taurine, methylhistidine, glycine, serine, creatine, alanine, glutamate, pyruvate, phosphatidylcholine (PTC), glycerophospholipids, and mono- and polyunsaturated fatty acids, together with the increase of diacylglycerol (DAG), butyrate, lysine, acetate, creatine phosphate, tyrosine, lactate, and formate, was observed, compared with controls. Conversely, after six-hour cisplatin exposure, no significant metabolic changes were found in the SH-SY5Y cell samples for both aqueous or lipid fractions. This result is in agreement with the known cisplatin action mechanism characterized by a genomic target, which requires longer exposure (about twelve hours) to induce relevant metabolomic changes [26, 27].

The further 24-hour monitoring comparison of the treatments (Pt-EtOMeSOphen, cisplatin) with respect to the controls showed a peculiar metabolite modulation behaviour for the novel Pt complex. Indeed, a marked decrease of PTC, glycerophospholipids, mono- and polyunsaturated fatty acids, glutathione, choline, and glycerophosphocholine associated with an increase of DAG and acetate was specifically observed only for the Pt-EtOMeSOphen treatment. The detected metabolic alterations strongly suggest a different mechanism of action for Pt-EtOMeSOphen compared with cisplatin.

**Figure 6:**
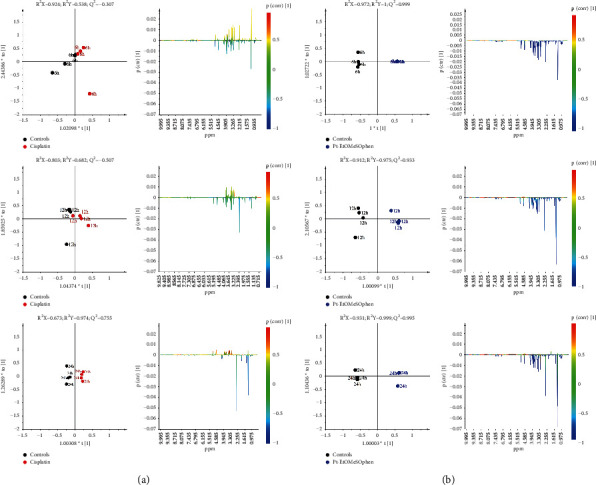
Time response pairwise comparison OPLS-DA score plot models (1 + 1 + 0 components) and corresponding color-coded correlation coefficient S-line plots displaying the related predictive discriminant loading variables derived from ^1^H NMR spectra of SH-SY5Y cell growth media obtained from different pairwise groups. (a) Controls vs. cisplatin. (b) Controls vs. Pt-EtOMeSOphen.

Further analysis was performed on the exo-metabolome (growth media) of treated cells. As observed from PCA (Figure 2(c) right panel), no significant differences between cisplatin and control growth media metabolic profiles were observed. This evidence was also confirmed from the supervised OPLS-DA analysis. Indeed, a low predictive ability (*Q*^2^ with negative values), and thus a low differentiation of the sample classes, was obtained for cisplatin-treated vs. control samples at six- and twelve-hour drug exposure (*Q*^2^ = −0.307 and *Q*^2^ = −0.507 respectively) (Figure 6(a)). A good separation between the growth media profiles of cisplatin-treated and untreated samples was observed only after twenty-four hours. In detail, at longer incubation times (24 h), the growth media of cisplatin-treated samples, in comparison with controls, was characterized by a general lower content of lactate, pyruvate, alanine, and acetate and higher glucose. On the other hand, Pt-EtOMeSOphen-treated cells' growth medium showed, in the OPLS-DA pairwise comparisons, a significant consumption of nutrients, compared with the controls, already evident after six hours (Figure 6(b)).

The reported full OPLS-DA pairwise comparison (Figure 6(b)) shows a marked consumption of nutrients (amino acids and glucose) and the decrease in the general content levels of some metabolic intermediates (acetate, pyruvate, lactate, formate) for Pt-EtOMeSOphen compared with controls, observed for all the incubation times. Pt-EtOMeSOphen exposure caused, already after six hours of exposure, a higher energy supply variations on the treated SH-SY5Y cells compared with controls. The lower levels of alanine, pyruvate, and glutamine in cell growth media (with respect to both controls and cisplatin), already observed at six hours after treatment, indicated early cell death-activated processes [20, 27].

### 3.3. Pathway Analysis

**Figure 7:**
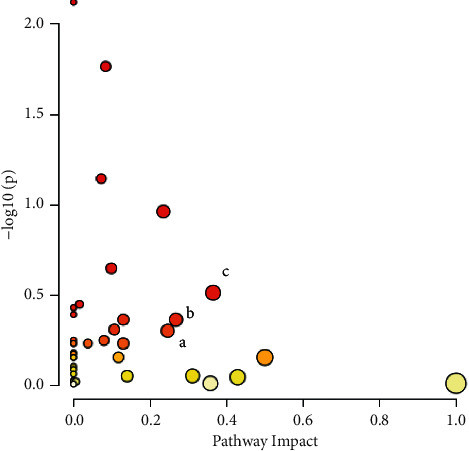
Summary of metabolic analysis conducted using MetaboAnalyst considering the 6 hours after treatment of Pt-EtOMeSOphen vs. controls. (a) Glycine, serine, and threonine metabolism. (b) Pyruvate metabolism. (c) Glutathione metabolism. Colors, varying from yellow to red, mean that the metabolites are in the data with different levels of significance (yellow: *p*-value <0.1; light orange: *p*-value <0.05; orange: *p*-value <0.01; and red: *p*-value <0.001). Circle size, varying from small to big, means the impact of metabolites in data on the pathways alteration (small circle: low impact and large circle: high impact).

Based on the observed metabolite variations previously discussed, we investigated in depth the six hour of Pt-EtOMeSOphen treatment results with respect to controls, showing the most relevant metabolic alterations. To reveal the most significant altered pathways associated with these latter, the metabolic pathway analysis, using MetaboAnalyst, was performed (Figure 7). The potential target pathways were determined considering both the significant metabolites (*p*-value <0.05) and the impact of the metabolites on the pathway alteration. Through this analysis, after the six hours of Pt-EtOMeSOphen treatment, glutathione metabolism, pyruvate metabolism, and the glycine, serine, and threonine metabolism emerged as potential involved pathways.

## 4. Discussion

In the present study, the metabolic fingerprinting of the effects induced by Pt-EtOMeSOphen, on the neuroblastoma cells SH-SY5Y, has been obtained by untargeted and targeted ^1^H NMR metabolomics investigation of cell lysates (aqueous and lipids extracts) and of growth media.

SH-SY5Y cells were treated at a concentration of Pt-EtOMeSOphen and cisplatin equal to their respective calculated at 24 h. Experimental exposure times of 6, 12, and 24 hours were considered. After treatments, cell lysates and growth media were analyzed through ^1^H NMR spectroscopy (Figure 2, left panel). Further multivariate unsupervised PCA (Figure 2, right panel) and supervised OPLS-DA analysis (Figures 3 and 4) were applied to the acquired ^1^H NMR spectra. According to the quantitative output offered by comparison of the bucket-reduced NMR spectra, the discriminant metabolites (for the endo- and exo-metabolome) identified from MVA were quantified and summarized as FC ratios in Figure 5. The performed multivariate data analysis defined a peculiar behaviour for Pt-EtOMeSOphen on the SH-SY5Y cells, in comparison with cisplatin, providing information about the mechanism of action and the possible cellular targets. After six hours of Pt-EtOMeSOphen exposure, metabolomic analysis revealed major changes in the metabolic profiles of SH-SY5Y cells, indicating a rapid response induced by the drug. The induction of rapid changes in the cellular metabolic profile (within six hours of treatment), following treatment with platinum-based anticancer drugs, has generally been associated with cytosolic cell targets [26]. These evidences strongly supported the hypothesis of a cytosolic target for the novel Pt-EtOMeSOphen compound. Consistently, also in the present study, only after twelve hours of treatment, cisplatin (which acts through a genomic target) showed appreciable alteration of cellular metabolite expression levels with respect to controls. Furthermore, from the metabolomics analysis, some metabolites were found as peculiar of the Pt-EtOMeSOphen action mechanism. In particular, the very low levels of expression of GSH in the Pt-EtOMeSOphen, with respect to both cisplatin-treated and untreated cells, were found to be of particular interest. Glutathione (GSH) is the prevalent low-molecular-weight thiol in mammalian cells [21]. Although in healthy cells the removal and detoxification of carcinogens is crucial, elevated GSH levels in tumor cells are associated with cancer progression and increased chemotherapeutic drug resistance. Indeed, recently, several novel therapies have been developed to target the GSH antioxidant system in tumors as a means for increased response and decreased drug resistance [36]. GSH is biosynthesized, starting from glutamate, cysteine, and glycine. The observed fast decrease of GSH levels in Pt-EtOMeSOphen-treated cells strongly supports the hypothesis of the glutathione metabolism as potential involved pathway responsible for the activity of this complex. This is also consistent with the observed high reactivity of Pt-EtOMeSOphen with thiol containing molecules, similar to that observed for other Pt complexes characterized by nongenomic targets in their action mechanism [37].

**Figure 8:**
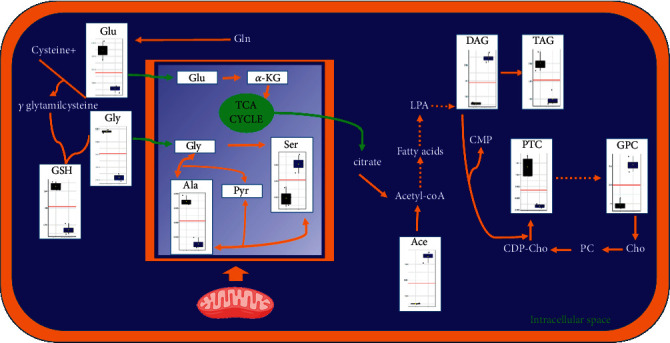
Schematic biochemical representation of the main metabolic pathways altered at six hours after Pt-EtOMeSOphen treatment. Box plots show the trend of highly significant altered metabolites (*p*value <0.01). Black: control cells; blue: Pt-EtOMeSOphen. Glu: glutamate, Gly: glycine, GSH: glutathione, *α*-KG: alpha-ketoglutarate, Ala: alanine, Pyr: pyruvate, Ser: serine, Ace: acetate, LPA: lysophosphatidic acid, DAG: diacylglycerol, TAG: triacylglycerol, PTC: phosphatidylcholines, GPC: glycerophosphocholine, Cho: choline, PC: phosphocholine, CDP-Cho: cytidine diphosphocholine.

Furthermore, after six hours of treatment, together with the decrease of GSH observed in aqueous cellular extracts, the accumulation of diacylglycerol (DAG) was also found, as specific of the Pt-EtOMeSOphen treatment in lipid extracts. This is in accord with literature data reporting lower activity for diacylglycerol acyltransferase (DAGT) and fatty acid synthase when glutathione is sequestered or absent [38]. DAGT is the enzyme responsible for the conversion of DAG to triacylglycerol (TAG). In the present study, generally lower content levels of TAG and higher levels of DAG were observed in Pt-EtOMeSOphen-treated samples, in comparison with controls and cisplatin. Therefore, in the present case, the increased DAG levels found may be explained by the low efficiency of DAGT to catalyze the synthesis of TGA. Interestingly, the DAG signal resonances are peculiar for Pt-EtOMeSOphen treatment since they are not clearly detectable in the NMR spectra of other lipid fractions (cisplatin treatment and controls). The proposed mode of action derived from the NMR-based metabolomics analysis is summarized in Figure 8 where box-and-whisker plots also illustrate the trend of significant metabolites grouped according to treatment and in comparison with control cells.

## 5. Conclusion

NMR-based metabolomics is a new field in the anticancer research and only recently has been applied in the study of the mechanism of action of metal-based drugs [24].

In this work, we explored the Pt-EtOMeSOphen mode of action on a neuroblastoma cancer cell line, SH-SY5Y cells, using a combination of NMR spectroscopy and pattern recognition data techniques. To obtain an overview of the whole changes induced by the drug, both endo- (aqueous and lipids extracts) and exo-metabolome (culture media) were analyzed. From the performed multivariate data analysis, a cytosolic target was strongly suggested for Pt-EtOMeSOphen and possibly attributable to some intermediate of the glutathione metabolism pathway. This aspect is very interesting since several novel therapies have been specifically designed to target the GSH antioxidant system in tumors as a means for increased pharmacological response and decreased drug resistance [36]. Indeed, GSH not only acts as a reducing agent and a major antioxidant within the cells maintaining a tight control of the redox status, but it is also a mediator of many other physiological reactions, including cellular signalling (involved in cell cycle regulation, proliferation, and apoptosis) [39]. In Pt-EtOMeSOphen-treated SH-SY5Y cells, the observed early (at 6 h) depletion of GSH, caused by the drug-induced stress, can also be associated with an early-induced apoptosis, as already reported [21, 39]. In the present case, the observed stress appears to be most likely due the thiol group reactivity toward the complex of GSH or some intermediates of its biosynthesis.

In conclusion, the NMR-based metabolomic analysis identified the effects of Pt-EtOMeSOphen on the SH-SY5Y cells metabolome, demonstrating a very different mechanism of action compared with cisplatin. The present study also confirms NMR metabolomics as an excellent tool to explore the mode of action of metal-based drugs.

## Data Availability

All data supporting the results are included in the article and supplementary file.

## Supplementary Materials

Supplementary Materials Figure S1. Expansion of Figure 2(a) left panel. Representative ^1^H NMR spectrum of SH-SY5Y cell aqueous extracts. Figure S2. Expansion of Figure 2(b) left panel. Representative ^1^H NMR spectrum of SH-SY5Y cell lipid extracts. Figure S3. Expansion of Figure 2(c) left panel. Representative ^1^H NMR spectrum of SH-SY5Y cell growth media. Table S1. Chemical shifts (*δ*, ppm) and assignments of metabolite resonances in the ^1^H NMR spectra of SH-SY5Y cell aqueous extracts and growth media. Table S2. Chemical shifts (*δ*, ppm) and assignments of metabolite resonances in the ^1^H NMR spectra of SH-SY5Y cell lipid extracts.
