# Supplementary material for: A NMR-Based Metabolomic Approach to Investigate the Antitumor Effects of the Novel [Pt(η1-C2H4OMe)(DMSO)(phen)]+ (phen = 1,10-Phenanthroline) Compound on Neuroblastoma Cancer Cells
Source: Bioinorg Chem Appl. 2022 Jun 10;2022:8932137. doi: 10.1155/2022/8932137 (PMC9205715; doi:10.1155/2022/8932137)
Supplement: Supplementary Materials — Figure S1. Expansion of Figure 2(a) left panel. Representative 1H NMR spectrum of SH-SY5Y cell aqueous extracts. Figure S2. Expansion of Figure 2(b) left panel. Representative 1H NMR spectrum of SH-SY5Y cell lipid extracts. Figure S3. Expansion of Figure 2(c) left panel. Representative 1H NMR spectrum of SH-SY5Y cell growth media. Table S1. Chemical shifts (δ, ppm) and assignments of metabolite resonances in the 1H NMR spectra of SH-SY5Y cell aqueous extracts and growth media. Table S2. Chemical shifts (δ, ppm) and assignments of metabolite resonances in the 1H NMR spectra of SH-SY5Y cell lipid extracts. [file 8932137.f1.docx]

**Supplementary Information**

NMR-based metabolomic approach to investigate the antitumor effects of the novel [Pt(η1-C_2_H_4_OMe)(DMSO)(Phen)]^+^ (Phen = 1,10-Phenanthroline) compound on neuroblastoma cancer cell line

Federica De Castro^1^, Erika Stefàno^1^, Erik De Luca^1^, Antonella Muscella^1^, Santo Marsigliante^1^, Michele Benedetti^1^*, Francesco Paolo Fanizzi^1^*

^1^ Department of Biological and Environmental Sciences and Technologies (DiSTeBA), University of Salento, Via Monteroni, I-73100 Lecce, Italy

Correspondence should be addressed to Michele Benedetti; [Michele.benedetti@unisalento.it](mailto:Michele.benedetti@unisalento.it) and Francesco Paolo Fanizzi; [fp.fanizzi@unisalento.it](mailto:fp.fanizzi@unisalento.it)

**Figure S1.** Expansion of Figure 2A left panel. Representative ^1^H NMR spectrum of SH-SY5Y cells aqueous extracts

**Figure S2**. Expansion of Figure 2B left panel. Representative ^1^H NMR spectrum of SH-SY5Y cells lipid extracts

**Figure S3**. Expansion of Figure 2C left panel. Representative ^1^H NMR spectrum of SH-SY5Y cells growth media

**Table S1.** Chemical shifts (δ, ppm) and assignments of metabolites resonances in the ^1^H NMR spectra of SH-SY5Y cells aqueous extracts and growth media.

**Table S2.** Chemical shifts (δ, ppm) and assignments of metabolites resonances in the ^1^H NMR spectra of SH-SY5Y cells lipid extracts.

**
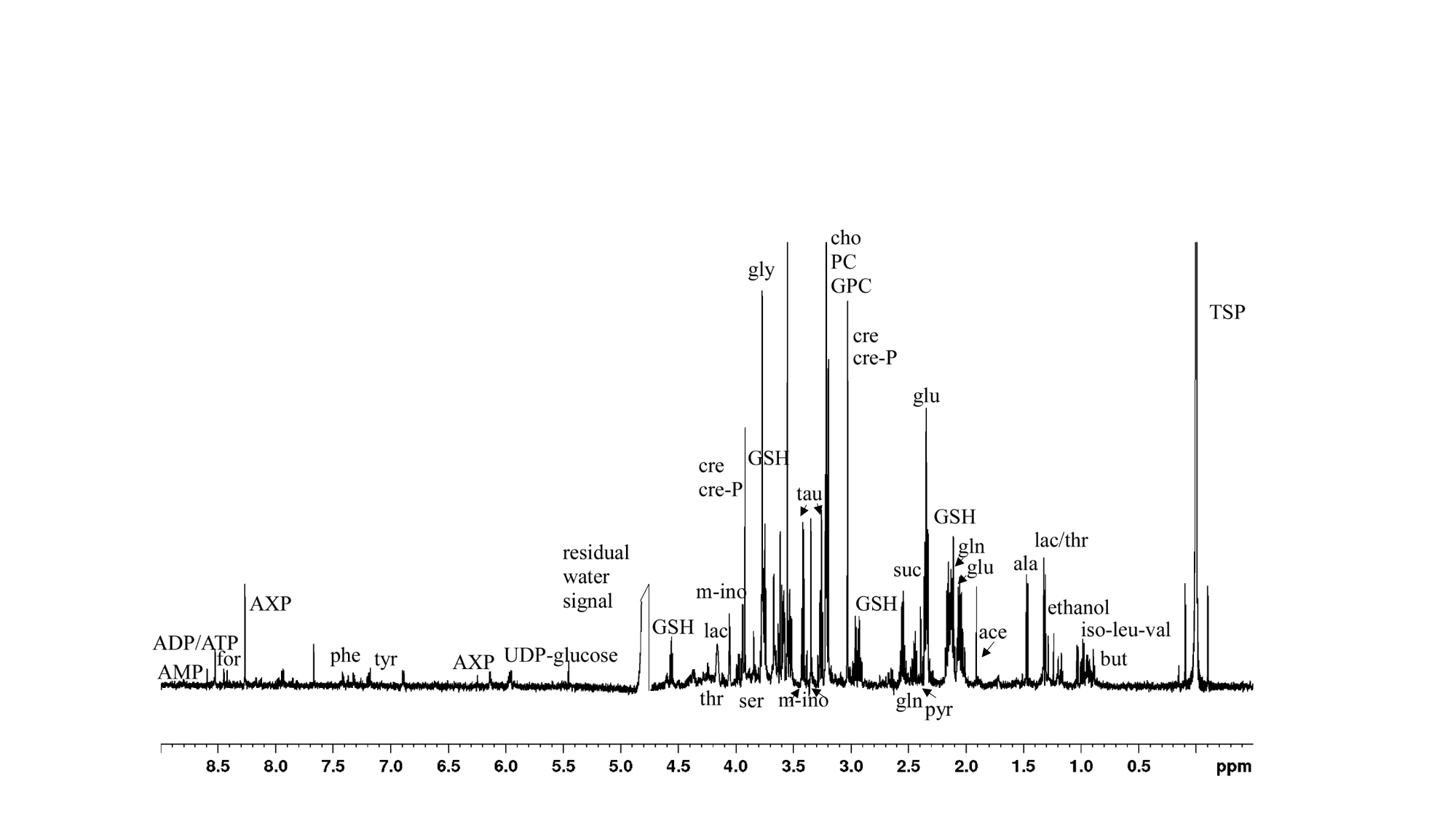
**

**Figure S1.**  Expansion of the figure 2A left panel. Representative 600 MHz ^1^H-NMR spectra of SH-SY5Y cells aqueous extracts.


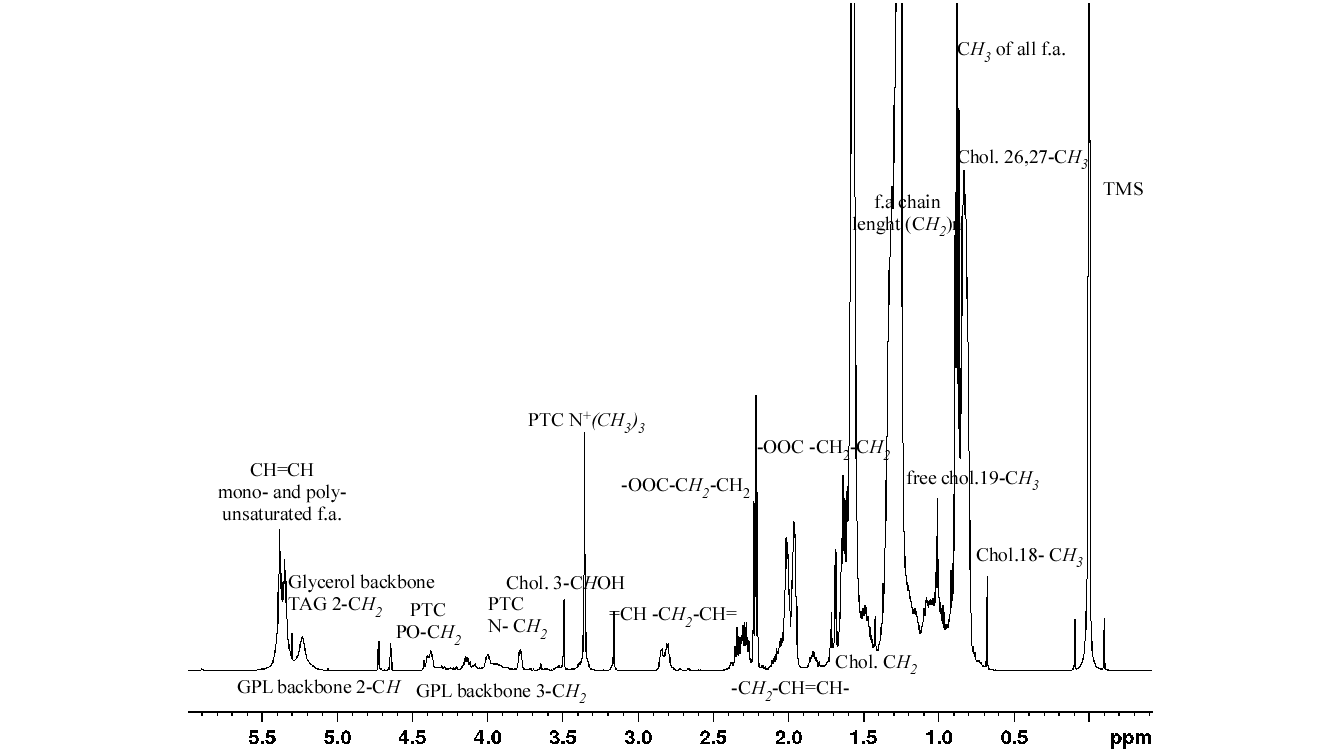


**Figure S2.** Expansion of Figure 2B left panel. Representative ^1^H NMR spectrum of SH-SY5Y cells lipid extracts.


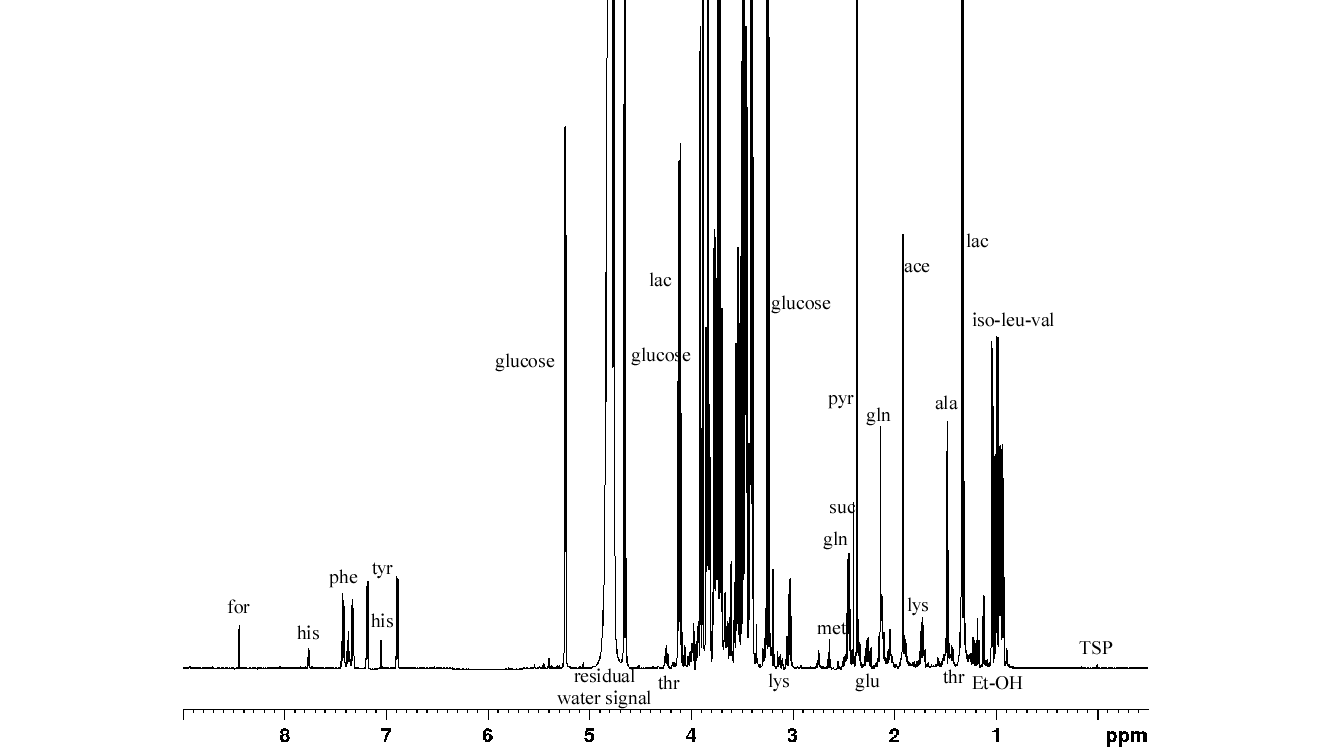


**Figure S3.** Expansion of Figure 2C left panel. Representative ^1^H NMR spectrum of SH-SY5Y cells growth media.

**Table S1.** Chemical shifts (δ, ppm) and assignments of metabolites resonances in the ^1^H NMR spectra of SH-SY5Y cells aqueous extracts and growth media.

| **Metabolites** | **^1^H NMR**  **chemical shifts**  **(δ, ppm)** | **Metabolites** | **^1^H NMR**  **chemical shifts**  **(δ, ppm)** |
| --- | --- | --- | --- |
| Alanine^(C,M)^ | 1.48(d)*; 3.78(q) | Butyrate ^(C)^ | 0.89(t)*; 1.57 (m); 2.15 |
| Creatine ^(C)^ | 3.04(s); 3.93(s)* | Creatine phosphate^(C)^ | 3.05(s); 3.94(s)* |
| Glutamate^(C,M)^ | 2.10(m); 2.35(m)*; 3.78(t) | Glutamine^(C,M)^ | 2.14(m);2.46(m)*; 3.78(t) |
| Glutathione^(C)^ | 2.15(m), 2.54(m)*, 2.97(dd), 3.78(m), 4.58(q) | Glycine^(C,M)^ | 3.56(s)* |
| Isoleucine^(C,M)^ | 0.94(t);1.01(d); 1.26(m);1.46(m); 1.98(m); 3.67(d) | Lactate^(C,M)^ | 1.33(d)*; 4.12(q) |
| Leucine^(C,M)^ | 0.96(d); 0.97(d);  1.70(m); 3.75(m) | Lysine^(C,M)^ | 3.02(t); 1.71*; 1.48; 1.91; 3.79 |
| Phenylalanine^(C,M)^ | 3.13(m);3.28(m); 4.00(m);7.33(d)*; 7.38(t); 7.43(m) | Serine ^(C,M)^ | 3.84(dd); 3.95(dd); 3.98(d)* |
| Taurine^(C)^ | 3.25(t); 3.43(t) | Threonine^(C,M)^ | 1.33(d);3.59(d); 4.25(m) |
| Tyrosine^(C,M)^ | 3.06(m);3.20(m); 3.94(m);6.92(d)*; 7.20(d) | Valine^(C,M)^ | 0.99(d); 1.04(d);  2.28(m); 3.61(d) |
| Choline^(C)^ | 3.19(s)*; 3.52(m); 4.07(m) | Glycerophosphocholine^(C)^ | 3.35(s);3.81(m); 4.30(m) |
| Phosphocholine^(C)^ | 3.20(s)*; 3.53(m); 4.14(m) |  |  |
| ­α-Glucose^(M)^ | 3.42(t);3.54(dd); 3.71(t);3.74(m); 3.84(m); 5.24(d)* | β-Glucose^(M)^ | 3.25(dd);3.41(t); 3.46(m);3.49(t); 3.72(dd); 3.90(dd);  4.65(d) |
| *myo*-Inositol^(C)^ | 3.25(t);3.61(dd); 4.07(t) |  |  |
| Acetate^(C,M)^ | 1.92(s)* | Formate^(C,M)^ | 8.46(s)* |
| Pyruvate ^(C,M)^ | 2.37(s)* | Succinate^(C,M)^ | 2.41(s)* |
| AMP^(C)^ | 6.15(d); 8.26(s) 8.59(s)* | ADP/ATP^(C)^ | 6.15(d); 8.26(s); 8.52(s)* |
| UDP-glucose^(C)^ | 5.98(d)*; 6.10(d) |  |  |

AMP: Adenosine monophosphate; ADP: Adenosine diphosphate; ATP: Adenosine triphosphate; UDP-glucose: uridine diphosphate-glucose. ^a^Letters in parentheses indicate the peak multiplicities; s, singlet; d, doublet; t, triplet; dd, doublet of doublet; q, quartet, m, multiplet. *Signals selected and integrated. ^(C)^Metabolites in HeLa cells; ^(M)^ Metabolites in cell growth medium.

**Table S2.** Chemical shifts (δ, ppm) and assignments of metabolites resonances in the ^1^H NMR spectra of SH-SY5Y cells lipid extracts.

| Metabolites | ^1^H NMR chemical shift (δ, ppm) | Metabolites | ^1^H NMR chemical shift (δ, ppm) |
| --- | --- | --- | --- |
| Cholesterol 18- C*H_3_* | 0.68(s) | Glycerol in 1-MAG 3′a-C*H_2_*–OCO- | 3.59 (dd) |
| Cholesterol 26,27- C*H_3_* | 0.87(d) | HO–C*H_2_*–CH–*sn*-1,2/2,3 DAG | 3.72 (dd) |
| C*H_3_* of all f.a. | 0.88(t)* | Phosphatidylcholine N- C*H_2_* | 3.83 (m) |
| free cholesterol 19-C*H_3_* | 1.01(t) | Glycerophospholipid backbone 3-C*H_2_* | 3.97(m) |
| f.a chain lenght (C*H_2_*)n | 1.28(m)* | C*H_2_*–OCO– *sn*-1,3 DAG | 4.13 (dd)* |
| Cholesterol C*H_2_* | 1.45-1.50(m) | C*H_2_*–OCO– *sn*-1,2 DAG | 4.23 (dd) |
| -OOC *-*CH_2_*-CH_2_* | 1.63(t) | Glycerolbackbone of TAG 1,3 C*H_2_* | 4.30 (dd)* |
| -C*H_2_*-CH=CH- | 1.96-2.15(m)* | Phosphatidylcholine PO-C*H_2_* | 4.41(m) |
| -OOC*-*C*H_2_*-CH_2_ | 2.24-2.44(m) | Glycerophospholipid backbone 2-C*H* | 5.22(m)* |
| =CH -C*H_2_*-CH= | 2.82 (m)* | Glycerol backbone of TAG 2-C*H_2_* | 5.26(m) |
| Phosphatydylcholine N^+^(C*H*_3_)_3_ | 3.34 (s)* | CH=CH of mono and poly-unsaturated f.a. | 5.35-5.40(m)* |
| Cholesterol 3-CHOH | 3.53 (m) |  |  |

MAG: monoacylglycerides; DAG: diacylglycerides; TAG: triglycerides; f.a.: fatty acids; ^a^Letters in parentheses indicate the peak multiplicities; s, singlet; d, doublet; t, triplet; m, multiplet. *Signals selected and integrated.
